# Long non-coding RNA OIP5-AS1 suppresses microRNA-92a to augment proliferation and metastasis of ovarian cancer cells through upregulating ITGA6

**DOI:** 10.1186/s13048-021-00937-3

**Published:** 2022-02-16

**Authors:** Yujue Wang, Lingling Li, Xun Zhang, Xiaolan Zhao

**Affiliations:** Gynaecology and Obstetrics Department, Sichuan Academy of Medical Sciences & Sichuan Provincial People’s Hospital, No. 32, West Second Section First Ring Rd, Chengdu, 610072 Sichuan China

**Keywords:** Ovarian cancer, Long non-coding RNA opa-interacting protein 5 antisense transcript 1, MicroRNA-92a, Integrin alpha 6, Proliferation, Metastasis

## Abstract

**Objective:**

Recently, long non-coding RNAs (lncRNAs) and microRNAs (miRNAs) have been identified as essential biomarkers during development of malignancies. This study was performed to study the roles of lncRNA opa-interacting protein 5 antisense transcript 1 (OIP5-AS1) and miR-92a in ovarian cancer (OC).

**Methods:**

OIP5-AS1, miR-92a and integrin alpha 6 (ITGA6) expression in OC tissues and cells was assessed. The screened OC cells were respectively with OIP5-AS1-, miR-92a- and ITGA6-related vectors or oligonucleotides . The viability, migration, invasion and apoptosis of the cells were determined and the levels of epithelial-mesenchymal transition (EMT)-related proteins were also measured. The interactions between OIP5-AS1 and miR-92a, and between miR-92a and ITGA6 were confirmed.

**Results:**

OIP5-AS1 and ITGA6 were upregulated while miR-92a was downregulated in OC. Inhibited OIP5-AS1 or downregulated ITGA6 or elevated miR-92a repressed EMT, viability, migration and invasion, and promoted apoptosis of OC cells. OIP5-AS1 as a competing endogenous RNA interacted with miR-92a to regulate ITGA6. These effects that induced by silenced OIP5-AS1 could be reversed by miR-92a inhibition while those that induced by up-regulated miR-92a were reduced by restored ITGA6.

**Conclusion:**

OIP5-AS1 silencing promoted miR-92a to repress proliferation and metastasis of OC cells through inhibiting ITGA6.

## Introduction

Ovarian cancer (OC) is the most fatal among all reproductive cancers, the 11st commonest type and the 5th major reason of cancer-related death in women [1]. It was estimated by the most recent global statistic that number of incident cases and deaths from OC has increased from 1990 to 2017 [2, 3]. This high mortality rate is induced by the absent or nonspecific symptoms in early stages of OC, resulting in delayed diagnoses until late stages [4]. In general, the initial treatments for OC are surgery and chemotherapy, which are effective for most patients. However, the recurrence may appear in OC patients within several years and treatment for recurrence is seldom curative [5]. Thus, novel targets for OC treatment are urgently needed.

Long noncoding RNAs (lncRNA) are functional RNA molecules containing over 200 nucleotides. LncRNAs modulate the expression of key genes via epigenetic modification and transcriptional and post-transcriptional regulation [6]. It has been identified that lncRNA HOTTIP acted as a predictive role in OC prognosis [7] and lncRNA LINC00152 promoted OC cell proliferation through regulating mitochondrial apoptosis pathways [8]. OPA-interacting protein 5 antisense transcript 1 (OIP5-AS1) situated at chromosome 15q15.1 and is evolutionarily conserved in vertebrates [9]. OIP5-AS1 has been revealed to participate in the progression of cervical cancer [10] and breast cancer [11]. Nevertheless, the role of OIP5-AS1 in OC remains rarely studied. It is known that lncRNAs are able to repress miRNA functions via serving as competing endogenous RNAs (ceRNAs) in human diseases [12]. MicroRNAs (miRNAs) are non-coding RNAs comprised of about 22 nucleotides, which post-transcriptionally functions on gene expression [13]. Some particular miRNAs have been reported to be implicated in OC. For instance, miR-203a-3p regulated the biological behaviors of OC cells [14], and miR-1307 affected the chemosensitivity of OC cells [15]. MiR-92a is one of the miRNAs that was considered to be related to the progression of OC [16] and aggressive breast cancer [17]. Nevertheless, the combined effect of OIP5-AS1 and miR-92a is still unexplored. Integrin alpha 6 (ITGA6) is a 150-kDa transmembrane protein [18] that has been revealed to be related to drug resistance and prognosis in OC [19].

We designed this research to investigate the role of OIP5-AS1/miR-92a/ITGA6 axis in OC, and we speculated that OIP5-AS1 may serve as a ceRNA to sponge miR-92a, thus regulating the biological processes of OC cells with the involvement of ITGA6.

## Materials and methods

### S2Ethics statement

Written informed consents were acquired from all patients before this study. The protocol of this study was confirmed by the Ethic Committee of Sichuan Academy of Medical Sciences & Sichuan Provincial People’s Hospital and based on the ethical principles for medical research involving human subjects of the Helsinki Declaration.

### S2Study subjects

OC tissues and adjacent normal tissues were harvested from 106 patients that accepted surgery in Sichuan Academy of Medical Sciences & Sichuan Provincial People’s Hospital and immediately preserved in liquid nitrogen for subsequent use. The primary OC was confirmed pathologically by veteran pathologists. Additionally, patients with other malignant diseases or previous neoadjuvant chemotherapy or radiotherapy were excluded [20, 21].

### S2Cell culture

Four OC cell lines (OVCAR3, SKOV3, A2780, and HO-8910) and human ovarian immortalized nontumorigenic ovarian surface epithelial cells (IOSE) were acquired from Procell Life Science Co., Ltd.,China and cultured in Dulbecco’s modified Eagle medium (DMEM) (TransGen Biotech Co., Ltd., Beijing, China) containing 10% fetal bovine serum (Gibco Company, NY, USA) and 1% penicillin/streptomycin (Sigma-Aldrich Chemical Company., MO, USA). All the cells were incubated in humidified chambers [21].

### S2Cell transfection

SKOV3 and A2780 cells were severally transfected with OIP5-AS1 silenced vector (sh-OIP5-AS1), miR-92a mimic/inhibitor, ITGA6 silenced vector (si-ITGA6), ITGA6 overexpression vector (oe-ITGA6) or their respective negative control (NC),sh-OIP5-AS1 and miR-92 inhibitor, sh-OIP5-AS1 + inhibitor NC, miR-92 mimic + oe-ITGA6, or miR-92a mimic + oe-NC. The above oligonucleotides and vectors were purchased from Genechem (Shanghai, China). The cells were cultured in 6-well plates until the cell confluence reached 70–80%, and then transfected with plasmids by Lipofectamine 2000 (Invitrogen, CA, USA) according to the protocols.

### S2Cell counting kit-8 (CCK-8) assay

The cell viability was determined in strict line with CCK-8 method (Dojindo Molecular Technologies Inc. Kumamoto, Japan). OC cells in each group were incubated on 96-well plates at 1000–3000 cells/well and the optical density was measured using a FLx800 Fluorescent Microplate Reader (BioTeke Corporation, Beijing, china).

### S2Colony formation assay

The colony formation ability of cells was assessed in accordance with CytoSelect™ 96-well cell transformation assay (standard Soft Agar kits, Cell Biolabs Inc. CA, USA). Cells in each group were seeded onto 96-well plates at 5000 cells/well and incubated for 2 w. The colonies were detected by CyQuant and analyzed by a FLx800 fluorescent microplate reader (Biotek).

### S2Transwell assay

Cells (2 × 10^5^) were suspended with 200 μL serum-free DMEM and seeded onto cell culture insert precoated with 1 μg/μL Matrigel. The basolateral chambers were appended with complete medium. Incubated for 48 h, the cells that did not penetrate through the membrane were removed, while the transmembrane cells were stained by 0.1% crystal violet dye solution. The numbers of invasive cells in five randomly fields were measured under a light microscope (Olympus Optical Co., Ltd., Tokyo, Japan).

### S2Scratch test

OC cells (5 × 10^5^) were seeded and covered the bottom of the plates on the second day. A 200 μL pipette tip was used to vertically scratched along the edge of a disinfected ruler (3 parallel scratches on each well). The supernatant was discarded and the wells were supplemented with serum-free medium to eliminate the effect induced by proliferation. Cells were photographed under a microscope after 24 h.

### S2Flow cytometry

Transfected cells were collected after 48 -h transfection. Fluoresceine isothiocyanate Annexin V Apoptosis Detection Kits (BD Biosciences, Franklin Lakes, NJ, USA) were utilized to assess the apoptosis on a FACScan Flow Cytometer. The apoptosis rate was calculated by Cell Quest software (BD Biosciences).

### S2Reverse transcription quantitative polymerase chain reaction (RT-qPCR)

Total RNA in tissues and cells was extracted by TRIzol kits (Invitrogen). A NanoDrop ND-3000 spectrophotometer (Life Technologies) was employed for RNA quantification and the RNA was reversely transcribed into cDNA. The expression of target genes were affirmed by SYBR Premix Ex Taq II kits (TaKaRa) and the StepOnePlus system (Applied Biosystems Inc., CA, USA). Glyceraldehyde phosphate dehydrogenase (GAPDH) was used as the standardized control of OIP5-AS1 and ITGA6, and U6 was used as the standardized control of miR-92a. Data were analyzed using 2^-ΔΔCt^ method and the primer sequences were shown in Table 1.

**Table 1:** Primer sequence used in this study

Gene	Primer sequence (5′-3′)
OIP5-AS1	F: 5′-TGCTGTGATGCTGGGAACTT-3′
	R: 5′-TGGGCTGATTGACCAATCTCA-3’
miR-92a	F: 5′-TATTGCACTTGTCCCGGCCTGT-3’
	R: mRQ 3’ Primer
ITGA6	F: 5′-GGCGGTGTTATGTCCTGAGTC-3’
	R: 5′-AATCGCCCATCACAAAAGCTC-3’
E-cadherin	F: 5′-CAGCATCACTGGCCAAGGAGCTGA-3’
	R: 5′-GACCACACTGATGACTCCTGTGTTCC-3’
Vimentin	F: 5′-CCGACACTCCT ACAAGATTTAGA-3’
	R: 5′-CAAAGATTTATTGAAGCAGAACC-3’
U6	F: 5′-GCTTCGGCAGCACATATACTAAAAT-3’
	R: 5′-CGCTTCACGAATTTGCGTGTCAT-3’
GAPDH	F: 5′-CGGAGTCAACGGATTTGGTCGTAT- 3’
	R: 5′-AGCCTTCTCCATGGTGGTGAAGAC-3’

*F* forward, *R* reverse, OIP5-AS1, opa-interacting protein 5 antisense transcript 1, miR-92a, microRNA-92a, ITGA6, integrin alpha 6, GAPDH, glyceraldehyde phosphate dehydrogenase, mRQ 3’ Primer were supplied with Mir-X™ miRNA First-Strand Synthesis and SYBR qRT-PCR kit (Takara Bio Inc., Japan)

### S2Western blot analysis

The proteins were extracted using radio-immunoprecipitation assay lysis buffer (Beyotime Biotechnology Co., Ltd., Shanghai, China). Equivalent proteins (20 μg) were separated by sodium dodecyl sulfate-polyacrylamide gel electrophoresis and transferred onto membranes for incubation with primary antibodies: ITGA6 (1: 10,000, Sigma) and GAPDH (1: 1000, Santa Cruz Biotechnology Inc., CA, USA). Subsequently, the cells were incubated with horseradish peroxidase-conjugated secondary antibody and the bands were developed by Pierce™ enhanced chemiluminescent Western Blotting Substrate (32,109, Thermo Fisher Scientific). The signal intensities of proteins were evaluated using Image J software (Promega, WI, USA).

### S2Dual luciferase reporter gene assay

Binding sites of OIP5-AS1 and miR-92a, miR-92a and ITGA6 were predicated by bioinformatics database RNA22 and TargetScan. The putative miR-92a target binding sequence in OIP5-AS1 and its mutant of the binding site was amplified by PCR and the PCR products were connected to pMirReporter plasmid (Sigma) to obtain OIP5-AS1 wild type (WT) vector (wt-OIP5-AS1) and OIP5-AS1 mutant type (MUT) vector (mut-OIP5-AS1). Similarly, ITGA6 WT (wt-ITGA6) and MUT (mut-ITGA6) report vectors were obtained. Afterwards, Lipofectamine 3000 reagent (Thermo Fisher Scientific) was utilized to transfect WT/MUT vectors and miR-92a mimic or mimic-NC into SKOV3 and A2780 cells on 96-well plates for 24 h. The luciferase activity was determined.

### S2RNA pull-down assay

The DNA fragment with OIP5-AS1 or its NC sequence was amplified by PCR through the T7-containing primer and bound to GV394 (Invitrogen). Plasmid DNA was digested with restriction enzyme XhoI and the biotin-labeled RNAs were reversely transcribed. Expression of target RNAs was determined by RT-qPCR [22].

### S2Statistical analysis

All data analyses were conducted using SPSS 21.0 software (IBM Corp. Armonk, NY, USA). The data were expressed as mean ± standard deviation. The t-test was performed for comparisons between two groups, analysis of variance (ANOVA) was used for comparisons among multiple groups and Tukey’s post hoc test was used for pairwise comparisons after one-way ANOVA. *P* value < 0.05 was indicative of statistically significant difference.

## Results

### S2OIP5-AS1 and ITGA6 are upregulated while miR-92a is downregulated in OC tissues

**Fig. 1:**
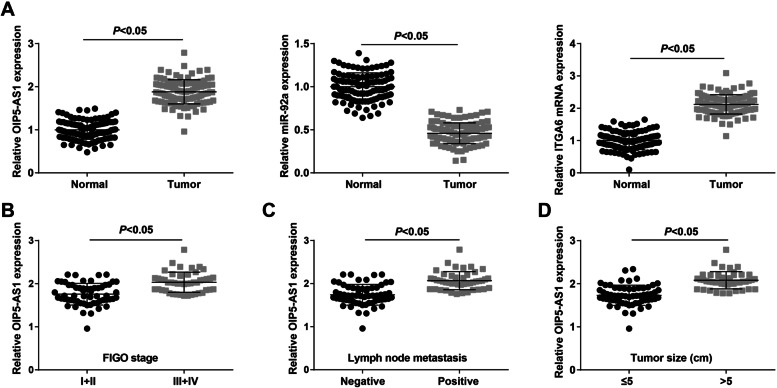
lncRNA OIP5-AS1 and ITGA6 are upregulated while miR-92a is downregulated in OC tissues; relationship between OIP5-AS1 expression and clinicopathological characteristics of OC patients. **A** OIP5-AS1, miR-92a and ITGA6 expression in OC tissues was detected by RT-qPCR; **B** relation between OIP5-AS1 expression and FIGO stage of OC patients; **C** relation between OIP5-AS1 expression and LNM of OC patients; **D** relation between OIP5-AS1 expression and tumor size of OC patients; A (*n* = 106), B, (I + II, *n* = 59; III + IV, *n* = 47), **C** (positive, *n* = 45; negative, *n* = 61), **D** (> 5, *n* = 45; ≤ 5, *n* = 61); the data were expressed as mean ± standard deviation and the t-test was performed for comparisons between two groups

OIP5-AS1, miR-92a and ITGA6 expression in clinical  tissues were gauged and we found significant higher expression of OIP5-AS1 and ITGA6, and lower expression of miR-92a in OC tissues (Fig. 1A).

To further explore the relation between OIP5-AS1 expression and clinicopathological characteristics of OC patients, we divided the OC tissues to the OIP5-AS1 high expression group (*n* = 53) and the OIP5-AS1 low expression group (*n* = 53) according to the median of OIP5-AS1 relative expression. The results suggested that OIP5-AS1 expression was higher in OC patients who were in an advanced International Federation of Gynecology and Obstetrics (FIGO) stage (Fig. 1B), lymph node metastasis (LNM) (Fig. 1C) and larger tumor size (Fig. 1D).

### S2Reduced OIP5-AS1 represses proliferation, migration, invasion and epithelial-mesenchymal transition (EMT) of OC cells, and accelerates cell apoptosis

**Fig. 2:**
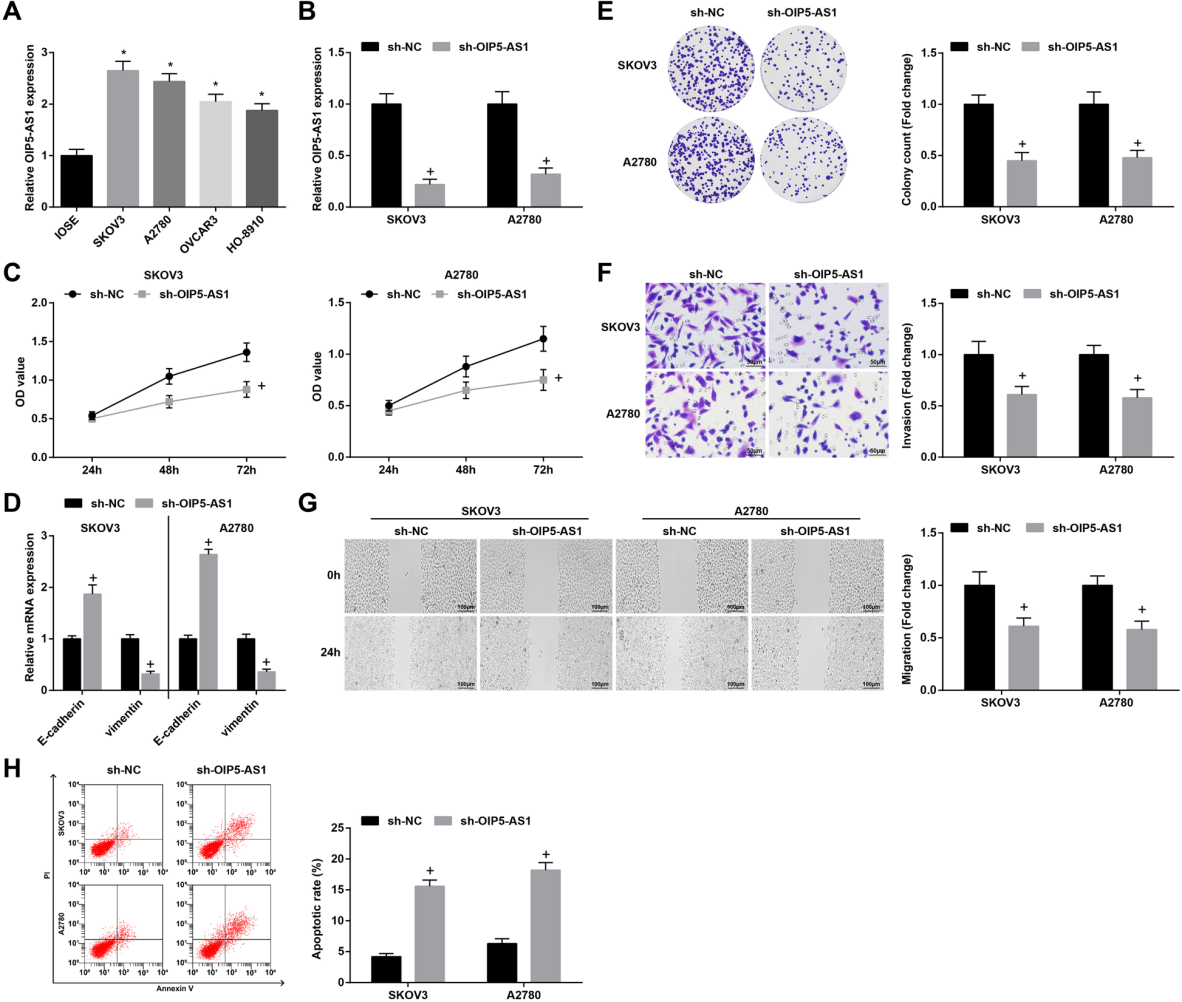
Reduced OIP5-AS1 represses proliferation, migration, invasion and EMT of OC cells, and accelerates cell apoptosis. **A-B** OIP5-AS1 expression in OC cell lines was assessed using RT-qPCR; **C** viability of SKOV3 and A2780 cells was detected by CCK-8 assay; **D** expression of EMT-related proteins in OC cells was measured by RT-qPCR; **E** colony formation ability of SKOV3 and A2780 cells was detected by colony formation assay; **F** Transwell assay was employed to determine the invasion ability of SKOV3 and A2780 cells; **G** migration ability of SKOV3 and A2780 cells was evaluated by scratch test; **H** apoptosis rates of SKOV3 and A2780 cells were measured by flow cytometry; * *P* < 0.05 vs IOSE, + *P* < 0.05 vs the sh-NC group; the data were expressed as mean ± standard deviation, t-test was performed for comparisons between two groups, ANOVA was used for comparisons among multiple groups

Higher OIP5-AS1 levels were observed in OC cells (OVCAR3, SKOV3, A2780, and HO-8910) than in human ovarian immortalized nontumorigenic ovarian surface epithelial cells IOSE (Fig. 2A). Additionally, SKOV3 and A2780 cells were selected for the subsequent assays. In SKOV3 and A2780 cells, transfection with sh-OIP5-AS1 reduced OIP5-AS1 expression (Fig. 2B). Experimentally, outcomes from CCK-8 assay, colony formation assay, Transwell assay and scratch test revealed that inhibition of OIP5-AS1 repressed viability, invasion and migration abilities, and also promoted apoptosis of OC cells. For EMT-related genes, RT-qPCR detection showed that E-cadherin was increased while that of Vimentin was decreased by reducing OIP5-AS1 (Fig. 2C-H).

### S2OIP5-AS1 interplays with miR-92a in a direct manner

**Fig. 3:**
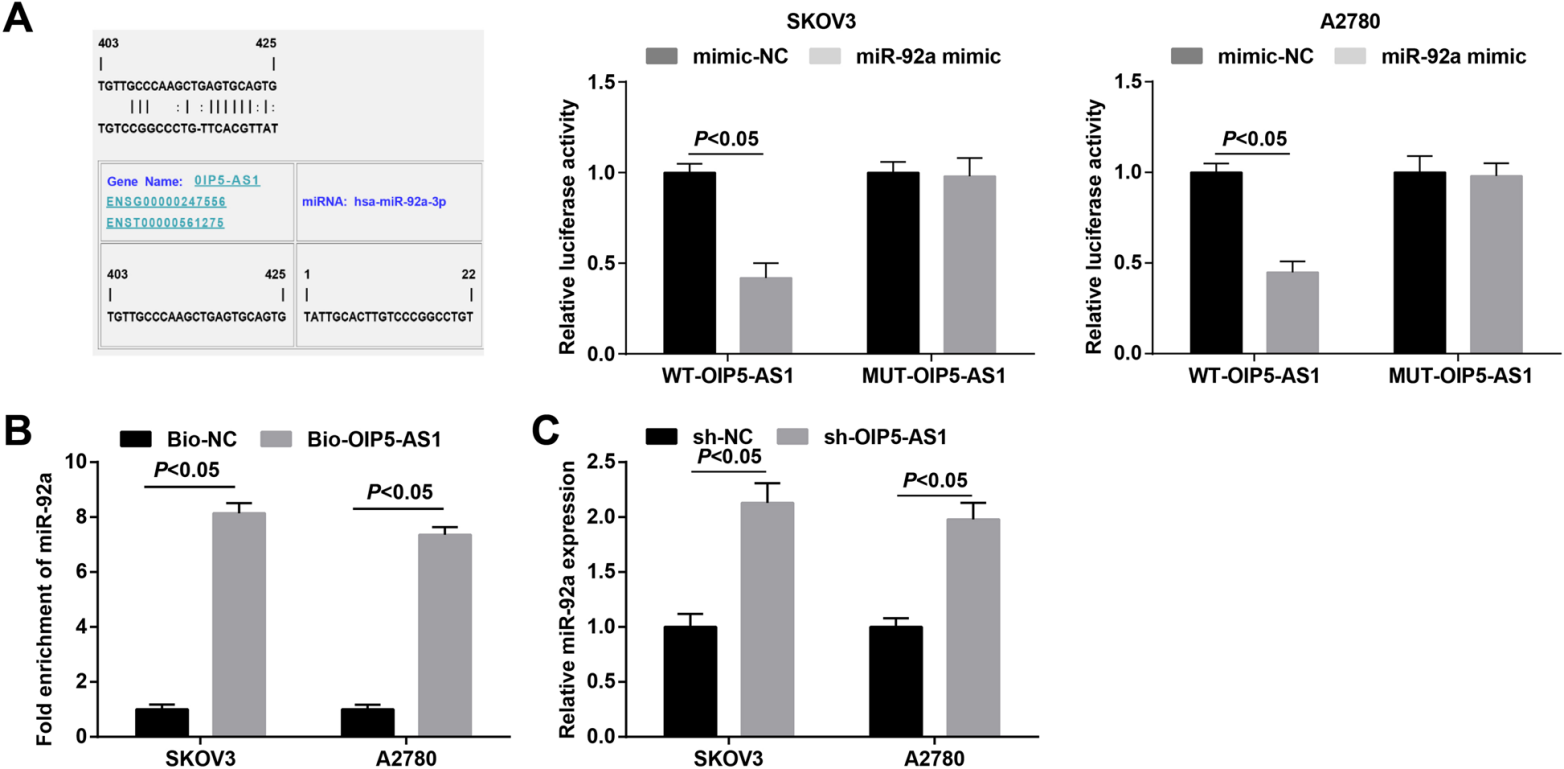
MiR-92a is a target of OIP5-AS1. **A** binding sites of miR-92a and OIP5-AS1 were predicted by a bioinformatic website and the relationship between miR-92a and OIP5-AS1 was confirmed by dual luciferase report gene assay; **B** miR-92a expression in samples pulled down by biotinylated OIP5-AS1 probe was detected by RT-qPCR; **C** expression of OIP5-AS1 and miR-92a in OC cells was determined by RT-qPCR; the data were expressed as mean ± standard deviation, t-test was performed for comparisons between two groups

The relation between miR-92a and OIP5-AS1 was evaluated by the bioinformatic website (https://cm.jefferson.edu/ra22/Precomputed/). It was further confirmed that miR-92a overexpression broadly inhibited the luciferase activity of wt-OIP5-AS1 (Fig. 3A). Subsequently, RNA pull-down assay was used to reveal that miR-92a was enriched in OC cells that had been pulled down by biotinylated OIP5-AS1 (Fig. 3B). Moreover, we found through RT-qPCR that miR-92a expression was raised after down-regulating OIP5-AS1 (Fig. 3C). These data showed that miR-92a directly bound to OIP5-AS1.

### S2Elevated miR-92a constrains proliferation, migration, invasion and EMT of OC cells, and accelerates cell apoptosis

**Fig. 4:**
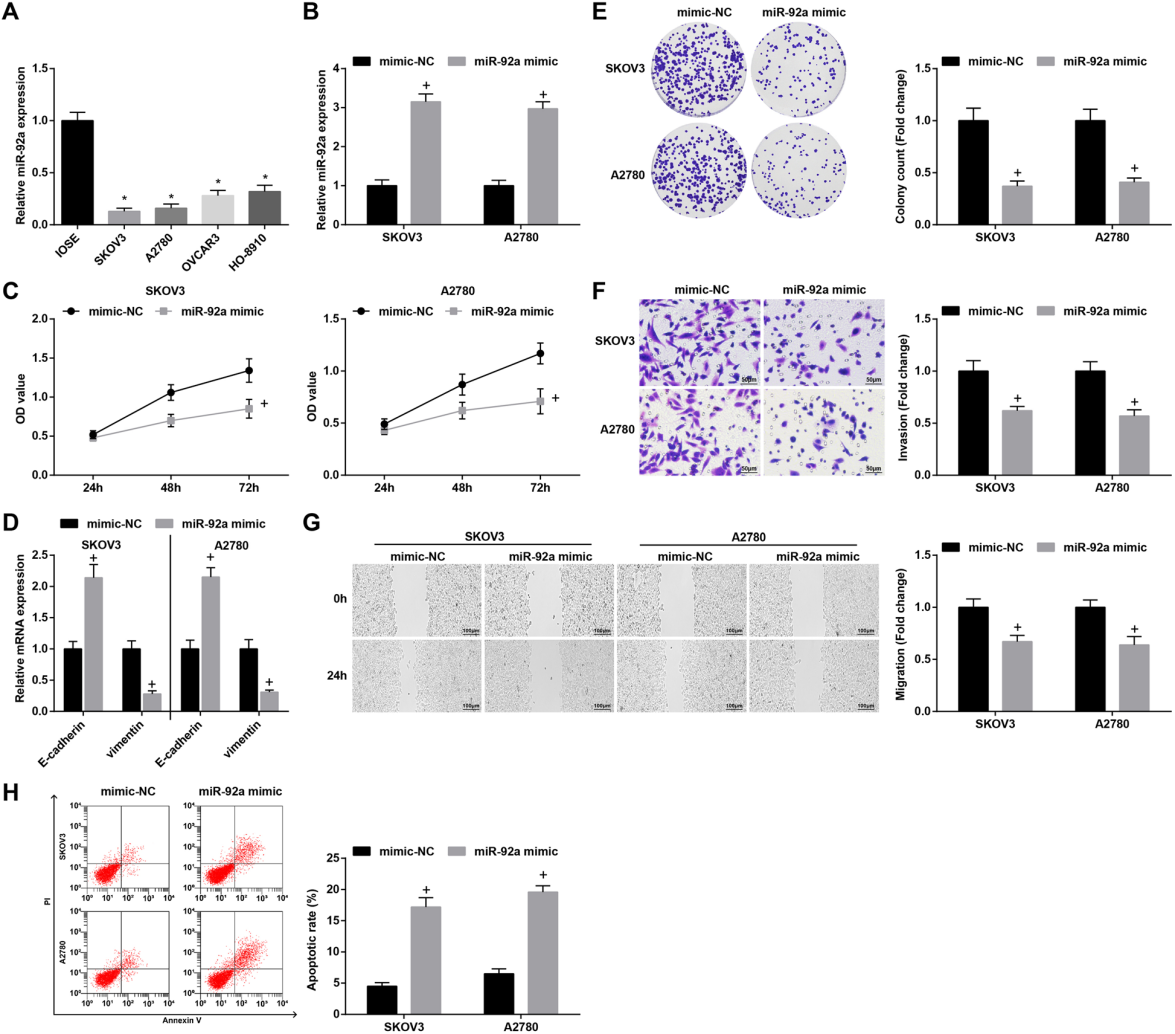
Elevated miR-92a constrains proliferation, migration, invasion and EMT of OC cells, and accelerates cell apoptosis. **A-B** miR-92a expression in OC cell lines was assessed using RT-qPCR; **C** viability of SKOV3 and A2780 cells was detected by CCK-8 assay; **D** expression of EMT-related proteins in OC cells was measured by RT-qPCR; **E** colony formation ability of SKOV3 and A2780 cells was detected by colony formation assay; **F** Transwell assay was employed to determine the invasion ability of SKOV3 and A2780 cells; **G** migration ability of SKOV3 and A2780 cells was evaluated by scratch test; **H** apoptosis rates of SKOV3 and A2780 cells were measured by flow cytometry; * *P* < 0.05 vs IOSE, + *P* < 0.05 vs the mimic-NC group; the data were expressed as mean ± standard deviation, t-test was performed for comparisons between two groups, ANOVA was used for comparisons among multiple groups

In SKOV3 and A2780 cells, transfection with miR-92a mimic elevated miR-92a expression (Fig. 4A-B). Furthermore, upregulation of miR-92a restrained viability, invasion and migration abilities, and also facilitated apoptosis of OC cells. Moreover, the mRNA expression of E-cadherin was heightened while that of Vimentin was lowered after promoting miR-92a expression (Fig. 4C-H).

### S2ITGA6 is targeted by miR-92a

**Fig. 5:**
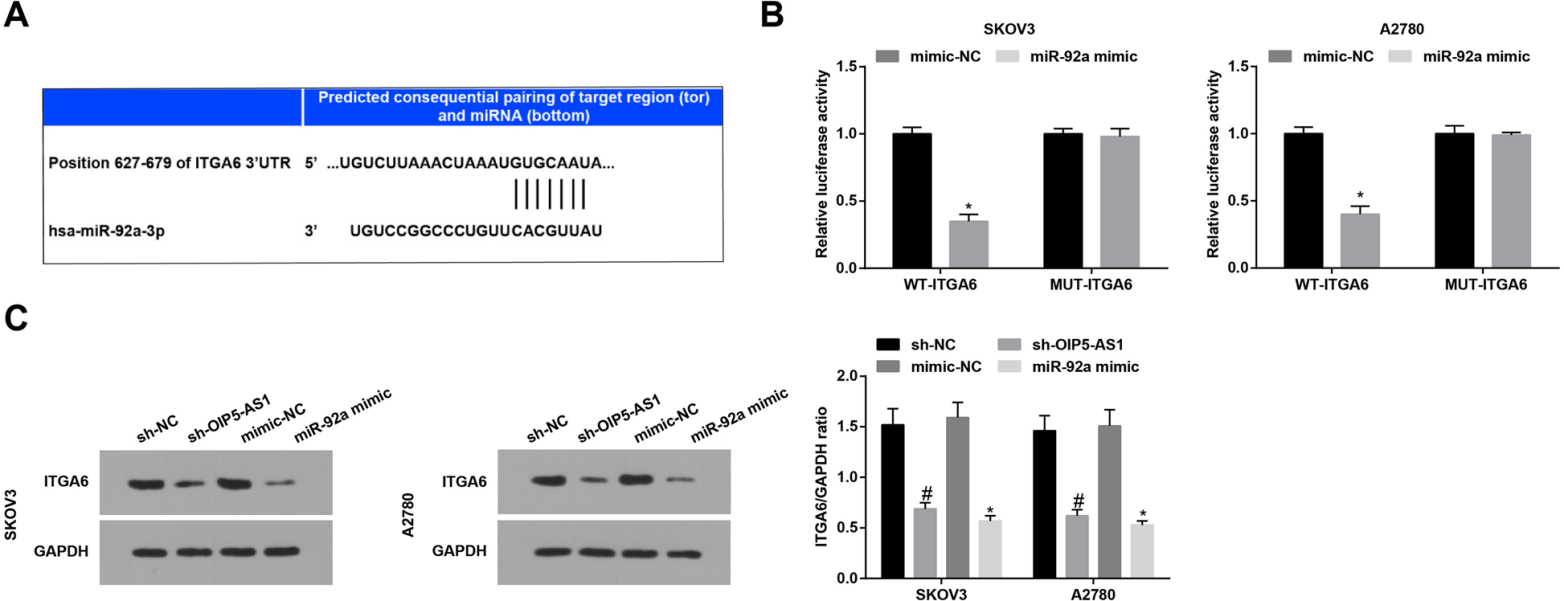
ITGA6 is targeted by miR-92a. **A-B** binding sites of miR-92a and ITGA6 were predicted by a bioinformatic website and the relationship between miR-92a and ITGA6 was confirmed by dual luciferase report gene assay; **C** ITGA6 expression in OC cells was assessed by Western blot analysis; * *P* < 0.05 vs the mimic-NC group, # *P* < 0.05 vs the sh-NC group; the data were expressed as mean ± standard deviation, t-test was performed for comparisons between two groups, ANOVA was used for comparisons among multiple groups

The bioinformatic website (http://www.targetscan.org/vert_72/) was applied to predict the target relation between miR-92a and ITGA6 (Fig. 5A). It was found in dual luciferase report gene assay that overexpression of miR-92a apparently repressed the activity of WT-ITGA6 3′-untranslated region (3’UTR) (Fig. 5B). Furthermore, we found via RT-qPCR and Western blot that silencing OIP5-AS1 or elevating miR-92a could reduce ITGA6 expression (Fig. 5C).

### S2Reduction of ITGA6 suppresses the malignancy of OC cells

**Fig. 6:**
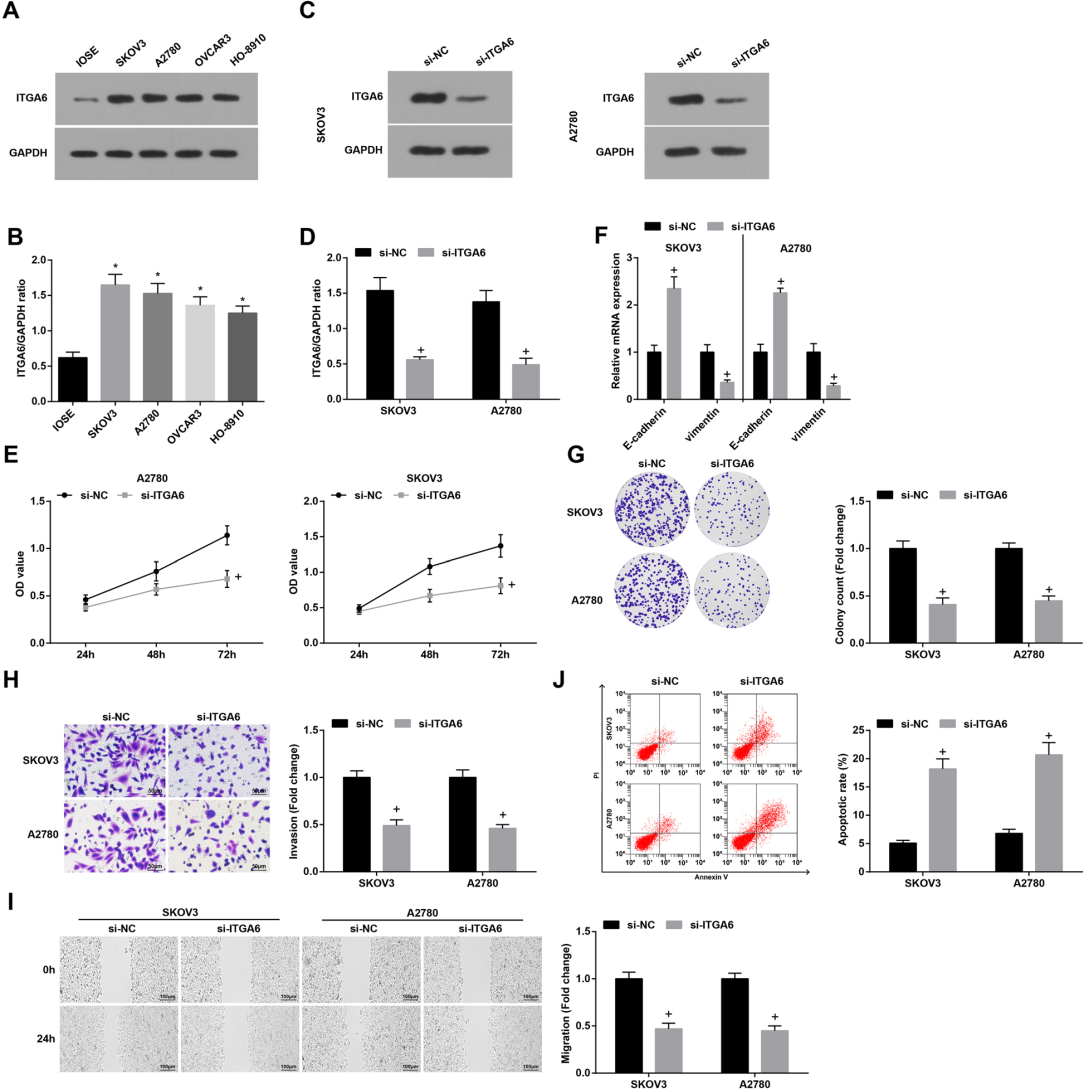
Reduction of ITGA6 suppresses the malignancy of OC cells. **A-B** ITGA6 expression in OC cells was detected by Western blot; **C-D** the transfection efficacy of si-ITGA6 was verified by Western blot; **E** viability of SKOV3 and A2780 cells was detected by CCK-8 assay; **F** expression of EMT-related proteins in OC cells was measured by RT-qPCR; **G** colony formation ability of SKOV3 and A2780 cells was detected by colony formation assay; **H** Transwell assay was employed to determine the invasion ability of SKOV3 and A2780 cells; **I** migration ability of SKOV3 and A2780 cells was evaluated by scratch test; **J** apoptosis rates of SKOV3 and A2780 cells were measured by flow cytometry; * *P* < 0.05 vs IOSE, + *P* < 0.05 vs the si-NC group; the data were expressed as mean ± standard deviation, t-test was performed for comparisons between two groups, ANOVA was used for comparisons among multiple groups

ITGA6 mRNA and protein expression was high in the four OC cell lines (Fig. 6A, B). It was observed in SKOV3 and A2780 cells that ITGA6 expression was knocked down after the treatment of si-ITGA6 (Fig. 6C and D). In answer to ITGA6 knockdown, SKOV3 and A2780 cells presented accelerated cell growth, elevated E-cadherin and reduced Vimentin mRNA expression (Fig. 6E-J).

### S2Inhibition of miR-92a reverses the effects of degraded OIP5-AS1 on biological processes of OC cells; overexpression of ITGA6 mitigates up-regulated miR-92a-mediated effects on the growth of OC cells

**Fig. 7:**
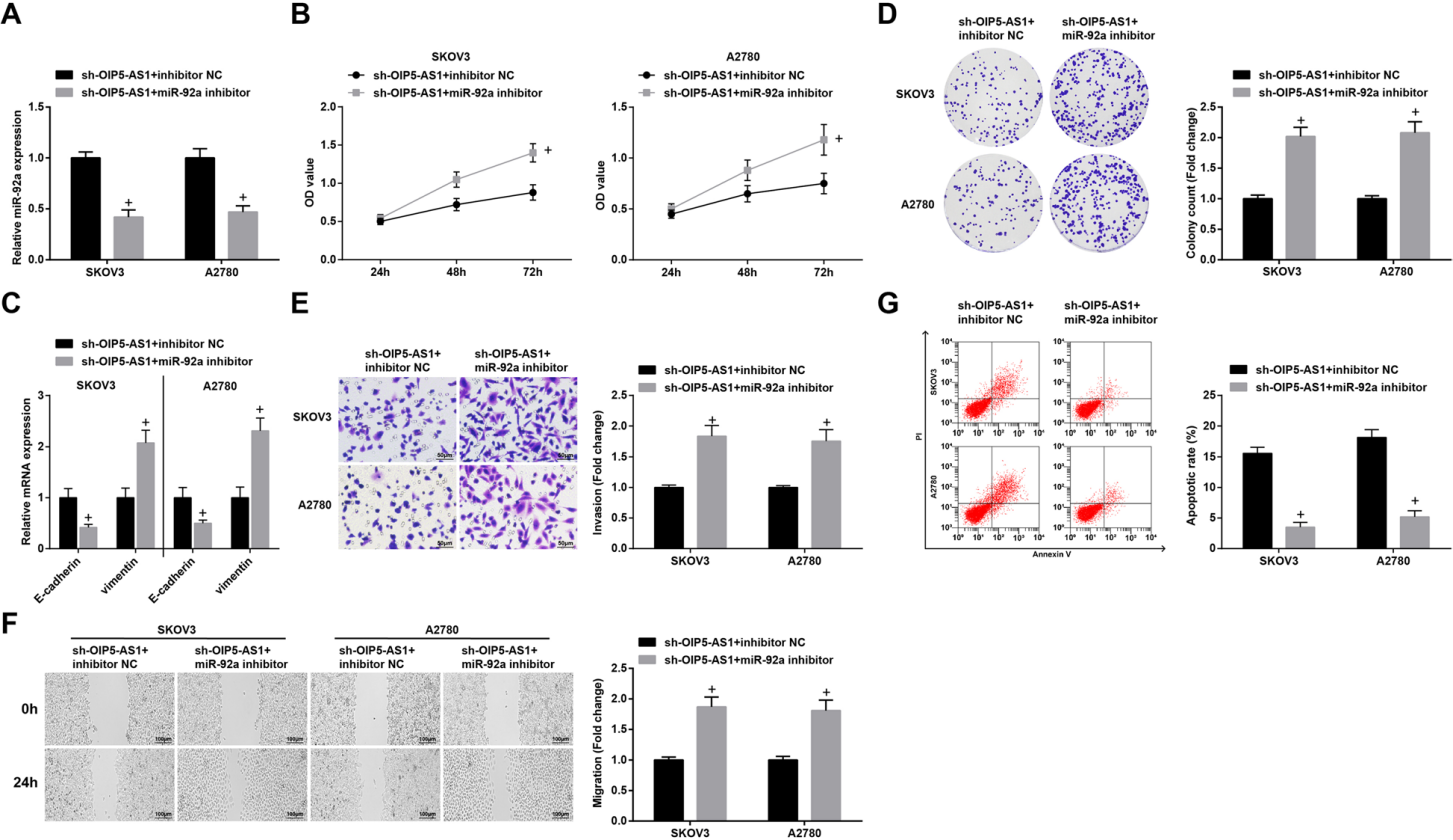
Inhibition of miR-92a reverses the effects of degraded OIP5-AS1 on biological processes of OC cells. **A** miR-92a expression SKOV3 and A2780 cells was detected by RT-qPCR; **B** viability of SKOV3 and A2780 cells was detected by CCK-8 assay; **C** expression of EMT-related proteins in OC cells was measured by RT-qPCR; **D** colony formation ability of SKOV3 and A2780 cells was detected by colony formation assay; **E** Transwell assay was employed to determine the invasion ability of SKOV3 and A2780 cells; **F** migration ability of SKOV3 and A2780 cells was evaluated by scratch test; **G** apoptosis rates of SKOV3 and A2780 cells were measured by flow cytometry; + *P* < 0.05 vs the sh-OIP5-AS1 group + inhibitor NC group; the data were expressed as mean ± standard deviation and the t-test was performed for comparisons between two groups

To explore the mechanism of OIP5-AS1 as ceRNA adsorbing miR-92a on OC cells, miR-92a and OIP5-AS1 were both silenced simultaneously by sh-OIP5-AS1 and miR-92a inhibitor. In SKOV3 and A2780 cells, we found that reduced miR-92a was able to reverse the impacts of OIP5-AS1 silencing on viability, migration, invasion and apoptosis of OC cells, as well as on E-cadherin and Vimentin mRNA expression (Fig. 7A-G).

**Fig. 8:**
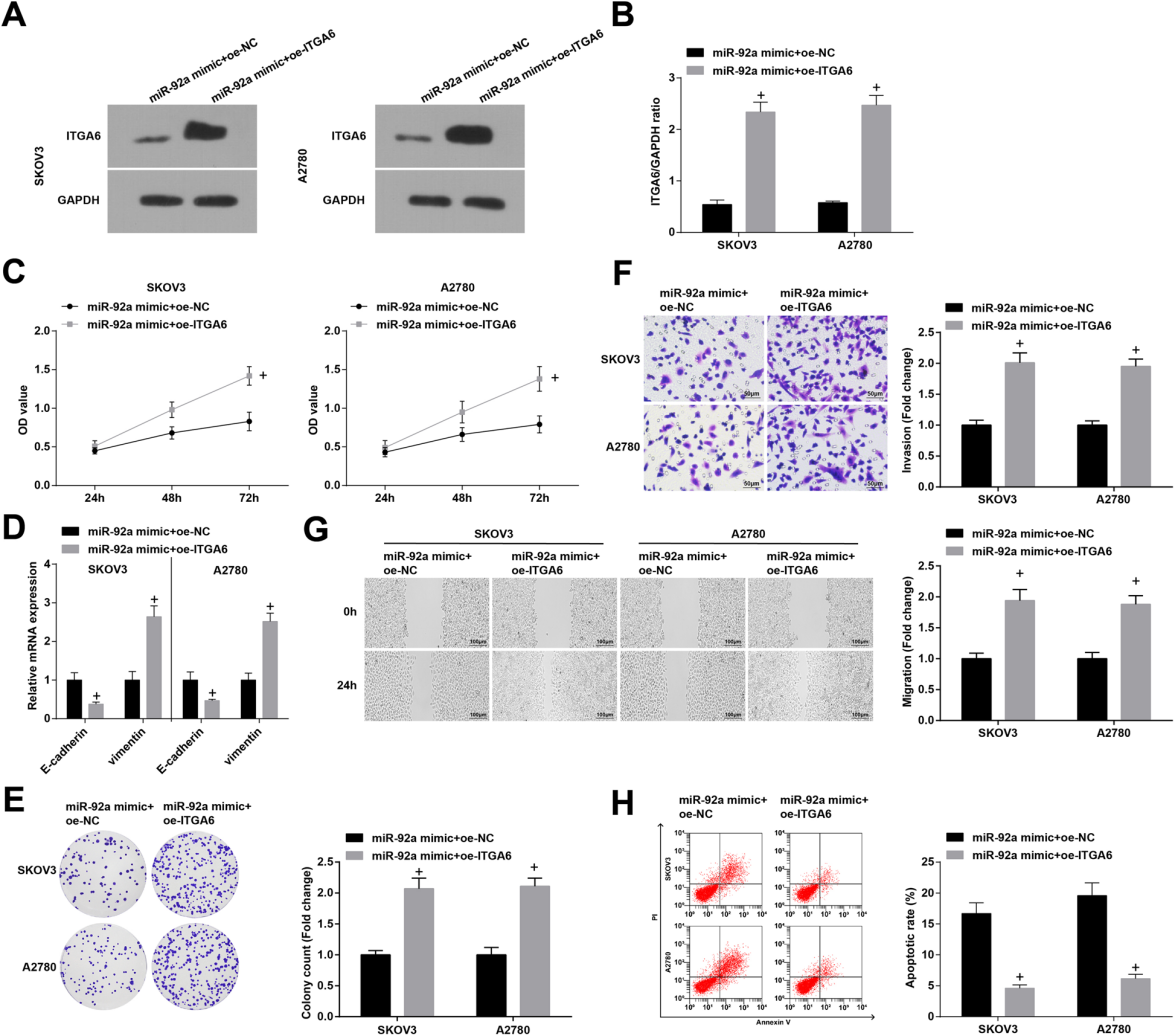
Overexpression of ITGA6 mitigates up-regulated miR-92a-mediated effects on the growth of OC cells. **A-B** ITGA6expression in SKOV3 and A2780 cells was detected by Western blot; **C** viability of SKOV3 and A2780 cells was detected by CCK-8 assay; **D** expression of EMT-related proteins in OC cells was measured by RT-qPCR; **E** colony formation ability of SKOV3 and A2780 cells was detected by colony formation assay; **F** Transwell assay was employed to determine the invasion ability of SKOV3 and A2780 cells; **G** migration ability of SKOV3 and A2780 cells was evaluated by scratch test; **H** apoptosis rates of SKOV3 and A2780 cells were measured by flow cytometry; + *P* < 0.05 vs the miR-92a mimic + oe-NC group; the data were expressed as mean ± standard deviation and the t-test was performed for comparisons between two groups

Also, we recognized that after up-regulation of ITGA6 by oe-ITGA6 (Fig. 8A and B), the inhibitory effects of miR-92a mimic on the growth and EMT process of OC cells were impaired (Fig. 8C-H).

## Discussion

OC is a malignancy that threatens women’s health globally. Although the incidence of OC is lower than that of cervical and endometrial cancers, its mortality is the highest among all gynecological cancers [23]. Accumulating evidence has shown that lncRNAs are capable of acting as ceRNAs to sponge miRNAs, thus modulating cell functions [24]. This research was performed to explore the role of OIP5-AS1 in the progression of OC by sponging miR-92a via regulating ITGA6, and the results of our experiments indicated that the reduction of OIP5-AS1 elevated miR-92a to repress proliferation and metastasis of OC cells through downregulating ITGA6.

To begin with, we determined the expression levels of OIP5-AS1, miR-92a and ITGA6 in tissues. It was found that OIP5-AS1 and ITGA6 were upregulated while miR-92a was downregulated in OC tissues. Consistently, Song et al. have unveiled that OIP5-AS1 was highly expressed in cervical cancer tissues [10]. A previous study has suggested that miR-92a was downregulated in breast cancer cells [17], and it has been identified that the expression of ITGA6 was increased in cisplatin-resistant SKOV3 and cisplatin-resistant A2780 cells, and also in drug-resistant tissues [19]. Next, the relationship between OIP5-AS1 and clinicopathological characteristics of OC patients has been analyzed and we discovered that the high expression of OIP5-AS1 was associated with advanced FIGO stage, LNM and larger tumor size of OC patients. Similarly, Yang et al. have affirmed that highly expressed OIP5-AS1 was related to advanced FIGO stage, LNM and poor overall survival of cervical cancer patients [25]. Moreover, we have found that OIP5-AS1 served as a ceRNA to absorb miR-92a, and ITGA6 was targeted by miR-92a. The regulatory relation between OIP5-AS1 and miR-92a remains unexplored while ITGA5 has been unraveled to be a target gene of miR-92a. However, the target relation of miR-92a and ITGA6 has not been studied yet.

Altered OIP5-AS1, miR-92a and ITGA6 were transfected into the OC cells to observe their roles in the biological behaviors of OC cells. The results of gain- and loss-of-function assays manifested that silenced OIP5-AS1 or elevated miR-92a restrained proliferation, migration and invasion of OC cells, and also promoted OC cell apoptosis. In accordance with our findings, Wang et al. have found that OIP5-AS1 promoted proliferation of lung cancer cells [26]. OIP5-AS1 has also been demonstrated to aggravate proliferation and migration of gastric cancer cells, and suppress while silenced OIP5-AS1 caused the opposite effects on tumor cells [27]. Currently, OIP5-AS1 has been determined to overexpress in epithelial OC and depletion of OIP5-AS1 contributes to limit cellular aggressiveness and EMT process [28]. Moreover, Q-Y Liu et al. have defined OIP5-AS1 with oncogenic property in OC and found that OIP5-AS1 knockdown represses OC cell viability, invasion, and migration [29]. A recent publication has uncovered that miR-92a-3p suppressed cell growth in Wilms’ tumor [30]. Gu et al. have also illuminated that miR-92a inhibited proliferation and induced apoptosis in acute myeloid leukemia [31]. Moreover, the expression of E-cadherin and vimentin was determined in our study, and we found that the knockdown of OIP5-AS1/ITGA6 or elevation of miR-92a heightened E-cadherin expression while lowered vimentin expression, indicating their repressive roles in EMT progression in OC. In line with these results, Wang et al. have pointed out that overexpressed OIP5-AS1 facilitated EMT of laryngeal squamous cell carcinoma cells [32], and reducing OIP5-AS1 has been verified to inhibit EMT progress in hepatoblastoma cells [33]. Furthermore, it has been recently discovered that miR-92b inhibited EMT in triple negative breast cancer cells [34] and nasopharyngeal cancer cells [35]. In addition, Zhang et al. have clarified that the oncogenic K-Ras upregulated ITGA6 could promote EMT [36].

To sum up, our study revealed that OIP5-AS1 elevated miR-92a to suppress proliferation and metastasis of OC cells by silencing ITGA6. This research may be helpful for exploring therapeutic strategies for OC. However, more efforts are required to investigate the detailed mechanisms.

## Data Availability

Not applicable
